# ZNF545 loss promotes ribosome biogenesis and protein translation to initiate colorectal tumorigenesis in mice

**DOI:** 10.1038/s41388-021-01938-8

**Published:** 2021-10-06

**Authors:** Shiyan Wang, Chi Chun Wong, Yanquan Zhang, Junzhe Huang, Chuangen Li, Jianning Zhai, Guoping Wang, Hong Wei, Xueji Zhang, Housheng Hansen He, Jun Yu

**Affiliations:** 1 Institute of Digestive Disease and Department of Medicine and Therapeutics, State Key Laboratory of Digestive Disease, Li Ka Shing Institute of Health Sciences, CUHK-Shenzhen Research Institute, The Chinese University of Hong Kong, Hong Kong, China; 2 Precision Medicine Institute, The First Affiliated Hospital, Sun Yat-sen University, Guangzhou, China; 3 School of Biomedical Engineering, Health Science Centre, Shenzhen University, Shenzhen, China; 4 Department of Medical Biophysics, Princess Margaret Cancer Centre, University Health Network, University of Toronto, Ontario, Canada

**Keywords:** Cancer genetics, Epigenetics

## Abstract

Ribosome biogenesis plays a pivotal role in tumorigenesis by supporting robust protein translation. We investigate the functional and molecular mechanism of Zinc finger protein 545 (ZNF545), a transcriptional repressor for ribosomal RNA (rRNA), in colorectal cancer (CRC). ZNF545 was silenced in CRC compared to adjacent normal tissues (*P* < 0.0001), implying a tumor-suppressive role. Colon-specific *Znf545* knockout in mice accelerated CRC in *Apc*^*Min/+*^ and azoxymethane/dextran sulfate sodium-induced CRC. Mechanistically, we demonstrated that ZNF545 uses its two zinc finger clusters to bind to minimal rDNA promoter, where it assembled transcriptional repressor complex by interacting with KAP1. *Znf545* deletion in mouse embryonic fibroblasts not only increased rRNA transcription rate and the nucleolar size and number but also altered the nucleolar composition and architecture with an increased number of fibrillar centers surrounded by net-like dense fibrillar components. Consequently, *Znf545* deletion promoted the gene expression of translation machinery, protein translation, and cell growth. Consistent with its tumor-suppressive role, ZNF545 overexpression in CRC cells induced growth arrest and apoptosis. Finally, administration of rRNA synthesis inhibitor, CX-5461, inhibited CRC development in *Znf545*^*Δ*/*Δ*^*Apc*^*Min/+*^ mice. In conclusion, ZNF545 suppresses CRC through repressing rRNA transcription and protein translation. Targeting rRNA biosynthesis in ZNF545-silenced tumors is a potential therapeutic strategy for CRC.

## Introduction

Colorectal cancer (CRC) is the third most common cancer worldwide and the third leading cause of cancer-related deaths [1]. Nevertheless, molecular mechanisms leading to CRC initiation and development remain elusive. The ribosome is a fundamental cellular component in living cells that mediates protein translation and consists of two major components: ribosomal RNA (rRNA) and ribosomal proteins, that are constructed within the nucleolus. rRNA performs critical functions in the ribosome by allowing protein synthesis to occur. While hundreds of copies of the rRNA gene (rDNA) are located in tandem arrays at five chromosome locations in the human genome, only a small portion of rDNAs are being actively transcribed and the rest are silenced with a condensed heterochromatic structure and repressive histone modifications [2]. Nucleolar hypertrophy and increased ribosome biogenesis are characteristic hallmarks of malignant cells [3]. In recent years, increasing evidence has shown that nucleolar alterations and ribosome biogenesis as the driver of cancer proliferation and transformation [3, 4]. However, very few studies have been conducted to investigate the causative link between ribosome biogenesis and tumorigenesis in vivo.

Krüppel-associated box zinc finger proteins (KRAB-ZFP) are the largest subgroup of the C2H2-type zinc finger protein (ZFP) family, which constitute the largest family (~350 genes) of transcriptional repressors in the human genome [5, 6]. Despite the abundance of KRAB-ZFP, little is known regarding their physiological functions. Nevertheless, emerging studies suggest that the deregulation of KRAB-ZFPs has divergent effects on tumorigenesis, including cell proliferation, differentiation, and apoptosis. Our previous studies implicate a role for ZNF545, a novel KRAB-ZFP member in repressing rRNA transcription [7, 8]. However, its potential role in CRC remains unknown. We, therefore, hypothesize that the deregulation of ZNF545 might contribute to CRC initiation and progression through modulating rRNA biosynthesis and ribosome biogenesis.

In this study, we examined the causality between the silencing of ZNF545 and CRC using whole-body and colon-specific *Znf545* knockout mice (Supplementary Fig. S1) and elucidated its molecular mechanisms of action. We highlighted the unique features of ZNF545 for rDNA binding and how it recruits corepressors to induce repressive histone modification on the rDNA promoter. Consequently, ZNF545 down-regulates ribogenesis and global protein translation, thereby suppressing colorectal tumorigenesis. Finally, we showed that ZNF545 silencing confers hypersensitivity to rRNA inhibition, thus implying a potential therapeutic strategy for ZNF545-silenced CRC.

## Results

### Knockout of *Znf545* accelerates colorectal tumorigenesis in azoxymethane (AOM)/dextran sulfate sodium (DSS)-treated mice and *Apc*^*Min/+*^ mice

*ZNF545* expression was significantly down-regulated in the primary CRC tumor tissues compared to the adjacent normal tissues in the TCGA CRC cohort and our cohort (*P* < 0.0001; Supplementary Fig. S2a). Down-regulation of ZNF545 in tumor samples was observed in 81% of paired CRC samples in our cohort (116/143). These data suggest that the silencing of ZNF545 occurs widely in CRC, inferring a tumor-suppressive role.

**Fig. 1:**
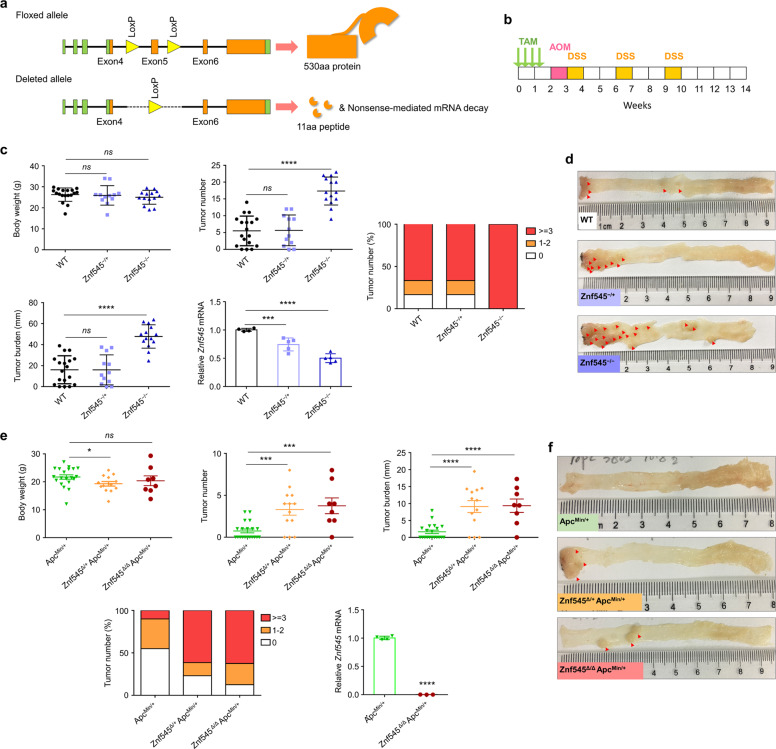
*Znf545* deficiency drives colon tumorigenesis in mice. **a** Schematic representation of the pre-conditional floxed allele and the deleted allele. Crossing mice bearing the floxed *Znf545* allele with mice expressing the Cre recombinase resulted in the removal of exon 5 flanked by the loxP sites and hence inactivated the *Znf545* gene. **b** Scheme for the experimental design of the AOM/DSS model. **c** The body weight, colon tumor number, colon tumor burden (WT: *n* = 18; colon-specific *Znf545*^*−/+*^: *n* = 12; *Znf545*^*−/−*^: *n* = 14), *Znf545* mRNA expression levels (WT: *n* = 4; colon-specific *Znf545*^*−/+*^: *n* = 5; *Znf545*^*−/−*^: *n* = 5) in colon tumors at the experimental endpoint in AOM/DSS model (WT: *n* = 18; colon-specific *Znf545*^*−/+*^: *n* = 12; *Znf545*^*−/−*^: *n* = 14). **d** Representative photographs of the intestines from WT, colon-specific *Znf545*^*−/+*^, and *Znf545*^*−/−*^ mice at the experimental endpoint in the AOM/DSS model. **e** The body weight, colon tumor number, colon tumor burden (*Apc*^*Min/+*^: *n* = 20; *Znf545*^*Δ/+*^*Apc*^*Min/+*^: *n* = 13; *Znf545*^*Δ/Δ*^*Apc*^*Min/+*^: *n* = 8) and *Znf545* mRNA expression levels (*Apc*^*Min/+*^: *n* = 4; *Znf545*^*Δ/Δ*^*Apc*^*Min/+*^: *n* = 3) in colon tumors of *Apc*^*Min/+*^, *Znf545*^*Δ/+*^*Apc*^*Min/+*^, *Znf545*^*Δ/Δ*^*Apc*^*Min/+*^ mice at 3 months of age in *Apc*^*Min/+*^ mice model. **f** Representative photographs of the intestines from *Apc*^*Min/+*^, *Znf545*^*Δ/+*^*Apc*^*Min/+*^, *Znf545*^*Δ/Δ*^*Apc*^*Min/+*^ mice at 3 months of age in *Apc*^*Min/+*^ mice model. All histogram data represent mean ± SD (two-sided Student’s *t* test). ns not significant; *P < 0.05; **P < 0.01; ***P < 0.001; ****P < 0.0001.

To test our hypothesis that ZNF545 might function as a tumor suppressor in colorectal tumorigenesis, we utilized two mouse models: AOM/DSS model and *Apc*^*Min/+*^ model. We generated colon epithelium-specific, inducible *Znf545* knockout mice (*Znf545*^*−/−*^) by crossing *Znf545*^*flox/flox*^ mice to *CDX2P-CreER*^*T2*^ mice (Supplementary Fig. S1). At 7 weeks of age, mice were given tamoxifen injection to activate Cre recombinase fusion protein CreER^T2^, leading to colon epithelium-specific excision of floxed exon 5 of *Znf545*. Cre-mediated excision of exon 5 results in a frameshift and a premature stop codon in exon 6, generating a short 11aa peptide and triggering nonsense-mediated mRNA decay (NMD) (Fig. 1a), in a similar approach as reported previously [9–12].

In the AOM/DSS model, colon epithelium-specific *Znf545*^*−/−*^ mice (*Znf545*^*flox/flox*^*CDX2P-CreER*^*T2*^), *Znf545*^*−/+*^ mice (*Znf545*^*flox/+*^*CDX2P-CreER*^*T2*^), and wild type (WT) mice (*CDX2P-CreER*^*T2*^) were treated with tamoxifen, followed by carcinogen AOM and three cycles of DSS treatment to induce colon cancer (Fig. 1b). Colon epithelium-specific *ZNF545* knockout had no effect on overall body weight (Fig. 1c). Whilst only 67% of *Znf545*^*−/+*^ and WT mice harbored multiple tumors (≥3), all the *Znf545*^*−/−*^ mice had multiple tumors (Fig. 1c,d; Supplementary Fig. S2b; Supplementary Table 1). Consistently, *Znf545*^*−/−*^ mice showed a significant increase in tumor number (*P* < 0.0001) and burden (*P* < 0.0001; the sum of all tumor size per mouse) compared to WT mice (Fig. 1c) [13].

For the *Apc*^*Min/+*^ model, whole-body *Znf545* knockout mice (*Znf545*^*Δ/Δ*^) was crossed with *Apc*^*Min/+*^, a model of spontaneous CRC, to generate *Znf545*^*Δ/Δ*^*Apc*^*Min/+*^, *Znf545*^*Δ/+*^*Apc*^*Min/+*^ and *Apc*^*Min/+*^ mice (Supplementary Fig. S1b). Similar to that in AOM/DSS model, *Znf545* whole-body knockout had a negligible influence on body weight (Fig. 1e). Macroscopic examination of the mice colon conducted at 3 months of age revealed that the majority of *Znf545*^*Δ/Δ*^*Apc*^*Min/+*^ (88%) and *Znf545*^*Δ/+*^*Apc*^*Min/+*^ (77%) mice developed colon tumors, whilst <50% of *Apc*^*Min/+*^ mice harbored visible tumors in the colon (Fig. 1e, f; Supplementary Fig. S2c; Supplementary Table 1). Both *Znf545*^*Δ/Δ*^*Apc*^*Min/+*^ and *Znf545*^*Δ/+*^*Apc*^*Min/+*^ mice demonstrated over fourfold increase in colorectal tumors compared to *Apc*^*Min/+*^ mice, both in terms of colon tumor number (*P* < 0.001) and burden (*P* < 0.0001) (Fig. 1e). Notably, heterozygous *Znf545* knockout was sufficient to promote tumor formation in *Apc*^*Min/+*^ mice but not in AOM/DSS model, indicating that different genetic backgrounds have differential susceptibility to ZNF545 silencing for tumorigenesis. Collectively, these findings confirmed that *Znf545* knockout promotes colorectal tumorigenesis in mice.

### ZNF545 exerts tumor-suppressive effects in CRC cell lines by inhibiting cell proliferation and inducing cell apoptosis

To assess the functional role of ZNF545 in CRC cells, we first examined its expression status in CRC cell lines. ZNF545 was silenced in 7 out of 8 CRC cell lines examined but strongly expressed in immortalized normal human colonic epithelial cell line HCEC 1CT and normal human colon tissues (Supplementary Fig. S3a), in consistency with the widespread down-regulation in CRC primary samples. Ectopic expression of ZNF545 in HT-29 and SW480 cells (Supplementary Fig. S3b) significantly suppressed colony formation (*P* < 0.0001) (Supplementary Fig. S3c) and induced cell apoptosis (*P* < 0.0001) (Supplementary Fig. S3d). Apoptosis was confirmed by the enhanced protein expression of apoptosis regulators, including cleaved poly (ADP-ribose) polymerase (PARP) and caspase-3, -7, and -8 in ZNF545-overexpressing HT-29 and SW480 cells compared with controls (Supplementary Fig. S3e). Consistent with knockout mice models, ZNF545 functions as a tumor suppressor in CRC cell lines.

### ZNF545 binds to the minimal rDNA promoter region

**Fig. 2:**
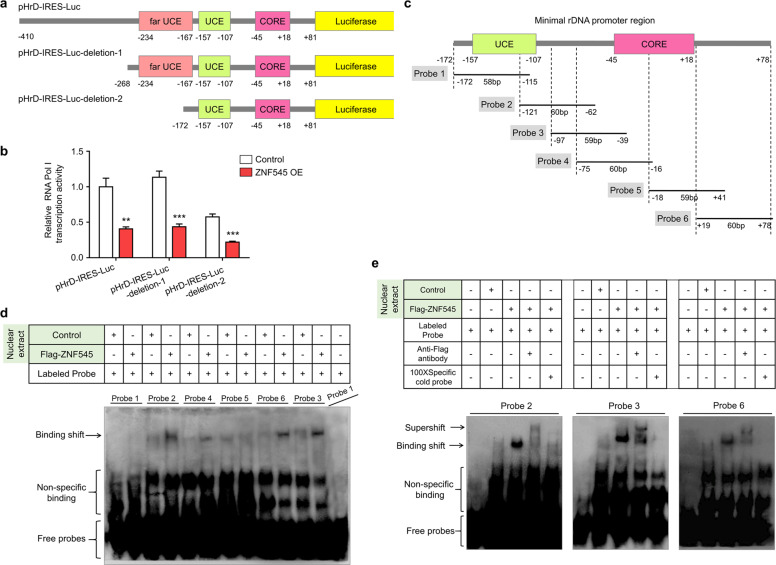
ZNF545 directly binds to the minimal rDNA promoter region. **a** Schematic representation of pHrD-IRES-Luc and two deletion constructs. **b** The effect of ZNF545 on rDNA promoter activity from pHrD-IRES-Luc and two deletion constructs assessed by dual-luciferase reporter assay (each group: *n* = 3). All histogram data represent mean ± SD (two-sided Student’s *t* test). Compared with control, **P < 0.01; ***P < 0.001. **c** Schematic representation of six overlapping biotinylated DNA probes spanning the minimal rDNA promoter region for EMSA. **d** Binding activity of the six biotinylated DNA probes to the nuclear extracts from Flag-tagged ZNF545-expressing and control HCT116 cells measured by EMSA. **e** EMSA competition and supershift assays of probes 2, 3, and 6. Competition reactions were performed by adding 100-fold unlabeled probes. Supershift reactions were performed by preincubation of nuclear extracts from Flag-tagged ZNF545-expressing HCT116 cells with anti-Flag antibody prior to the addition of the biotinylated DNA probes.

Since ZNF545 modulates rRNA transcription [7], we next assessed the potential binding sites of ZNF545 on the rDNA promoter. We constructed serial deletion mutants based on the pHrD-IRES-Luc construct containing human rDNA promoter (−410 to +81) and determined the impact of ZNF545 on luciferase activity (Fig. 2a) [14]. The minimal human rDNA promoter (−157 to +18) has a bipartite structure consisting of two distinct cis-control sequences, the core promoter element (CORE) proximal to initiation site (−45 to +18), and upstream control element (UCE) (−157 to −107) [15–17]. These are essential elements of rDNA promoter recognized by the upstream binding factor (UBF), which in turn recruits the selectivity factor (SL1) together with RNA polymerase I (Pol I) associated factors to transcription start site to initiate pre-rRNA synthesis [18–20]. Besides, the far UCE (−234 to −167) serves to modulate the efficiency of rRNA transcription [21]. In this regard, two deletion constructs, pHrD-IRES-Luc-deletion-1 (−268 to +81) and pHrD-IRES-Luc-deletion-2 (−172 to +81) were constructed (Fig. 2a). Deletion of sequences from −410 to −268 had no effect on luciferase activity, consistent with the previous results [21]. The further deletion of the far UCE decreased the basal transcriptional activity of the rDNA promoter by half (Fig. 2b) [21]. Nevertheless, ZNF545 overexpression significantly suppressed the transcription on all rDNA promoter-reporter deletion constructs (Fig. 2b), indicating that binding site(s) of ZNF545 should be present within the minimal rDNA promoter region.

### ZNF545 has two binding sites within the minimal rDNA promoter region

To further explore the binding site(s) of ZNF545, we performed the electrophoretic mobility shift assay (EMSA) using six ~60 bp overlapping biotinylated DNA probes spanning the minimal rDNA promoter region (Fig. 2c). Probe 2, 3, and 6 exhibited strong binding to Flag-tagged ZNF545 derived from nuclear extracts from Flag-tagged ZNF545 expressing cells (Fig. 2d). The specificity of the ZNF545 binding band of probes 2, 3, and 6 was confirmed by both competition and supershift assays (Fig. 2e). Since probes 2 and 3 shared overlapping sequences (Fig. 2c), one of the ZNF545 binding sites probably located within this overlapping area (−97 to −62). Moreover, ZNF545 was capable of binding to probe 6 but not probe 5, inferring that the other one of the ZNF545 binding sites should be contained within the non-overlapping sequences from probes 5 and 6 (+41 to +78). Taken together, there were two ZNF545 binding sites within the minimal rDNA promoter region: one was within the linking region between UCE and core promoter element, and the other was located downstream of the transcription start site.

### The binding of ZNF545 to the rDNA promoter requires its two zinc finger clusters

**Fig. 3:**
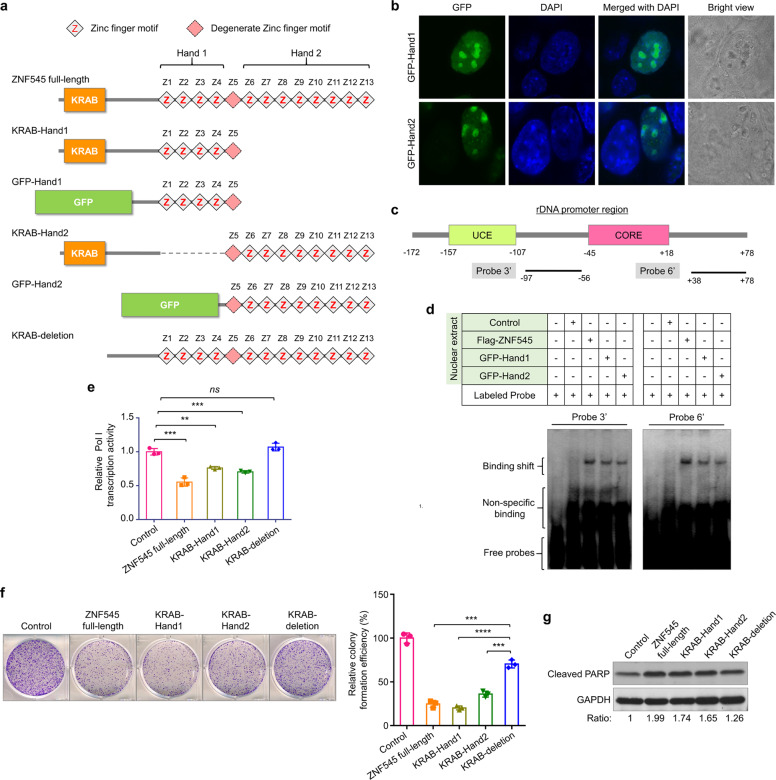
Molecular functions of the two zinc finger clusters and KRAB domain of ZNF545 protein. **a** Schematic representation of ZNF545 and protein variants. **b** Subcellular localization of GFP-Hand1 and GFP-Hand2 in HCT116 cells. **c** Schematic representation of the 2 biotinylated DNA probes (probe 3′ and probe 6′) for EMSA. **d** Binding activity of biotinylated probe 3′ and probe 6′ to the nuclear extracts from ZNF545-, GFP-Hand1- and GFP-Hand2-expressing and control HCT116 cells measured by EMSA. **e** The effect of ZNF545 and protein variants on rDNA promoter activity from pHrD-IRES-Luc construct assessed by dual-luciferase reporter assay (each group: *n* = 3). Effect of ZNF545 and protein variants overexpression on colony formation (**f**; each group: *n* = 3) and protein expression of cleaved PARP (**g**) in HCT116 cells. All histogram data represent mean ± SD (two-sided Student’s *t* test). ns not significant; **P < 0.01; ***P < 0.001; ****P < 0.0001.

ZNF545 protein contains twelve C2H2 zinc fingers arranged in two distinct clusters which are separated by a degenerate zinc finger motif (Z5): the first cluster, referred to as “Hand 1”, containing zinc fingers 1 to 4, and the second cluster in the C-terminus, referred to as “Hand 2”, containing zinc fingers 6 to 13 (Fig. 3a). All ZNF545 zinc fingers including degenerate zinc finger motif (Z5) are located in tandem repeats with consistent span and interval. C2H2 zinc finger motif makes sequence-specific recognition of DNA by contacting four or more bases to yield an overlapping pattern of contacts with adjacent zinc fingers [22]. However, in most cases, only part of the multiple adjacent zinc fingers participates in high-affinity DNA interaction [22]. In this regard, fusion proteins GFP-Hand1 and GFP-Hand2 were constructed by fusing GFP with “Hand 1” and “Hand 2” of ZNF545, respectively (Fig. 3a). Both GFP-Hand1 and GFP-Hand2 displayed a nucleolar localization (Fig. 3b), indicating that both of the zinc finger clusters harbor nucleolar localization signals and potentially bind to the rDNA promoter [23]. To test this possibility, EMSA was performed to evaluate DNA binding activity for each of ZNF545 zinc finger clusters using two biotin-labeled DNA probes (probe 3′ and probe 6′) encompassing the regions identified above (Fig. 3c). We revealed that both the GFP-Hand1 and GFP-Hand2 were capable of binding to the two probes in a similar manner to full-length ZNF545 (Fig. 3d), implying that both zinc finger clusters can bind to the rDNA promoter.

### Either of ZNF545 zinc finger clusters in conjunction with the KRAB domain represses rRNA transcription and tumorigenesis

As “Hand 1” and “Hand 2” bound to rDNA promoters independently of each other, we determined whether either of the zinc finger clusters coupled to transcriptional repression KRAB domain (KRAB-Hand1 or KRAB-Hand2) retains the rRNA suppressive effect of full-length ZNF545 (Fig. 3a). Indeed, luciferase assay demonstrated that both KRAB-Hand1 and KRAB-Hand2 repressed Pol I transcription activity, albeit to a lesser extent compared to full-length ZNF545 (Fig. 3e). In contrast, ZNF545 with the deletion of the KRAB domain (KRAB-deletion) failed to suppress Pol I transcription (Fig. 3e). Consistently, the overexpression of either KRAB-Hand1 or KRAB-Hand2 suppressed the growth of CRC cells in vitro (Fig. 3f), concomitant with induction of apoptosis as indicated by increased cleaved PARP expression (Fig. 3g). Hence, both of the zinc finger clusters play a critical role in homing of ZNF545 to rRNA promoter, where the KRAB domain can recruit its corepressors and exert its repressive effect on rRNA transcription, leading to tumor suppression.

### ZNF545 regulates histone modification on rDNA promoter by cooperating with corepressor KAP1 to block rRNA transcription

**Fig. 4:**
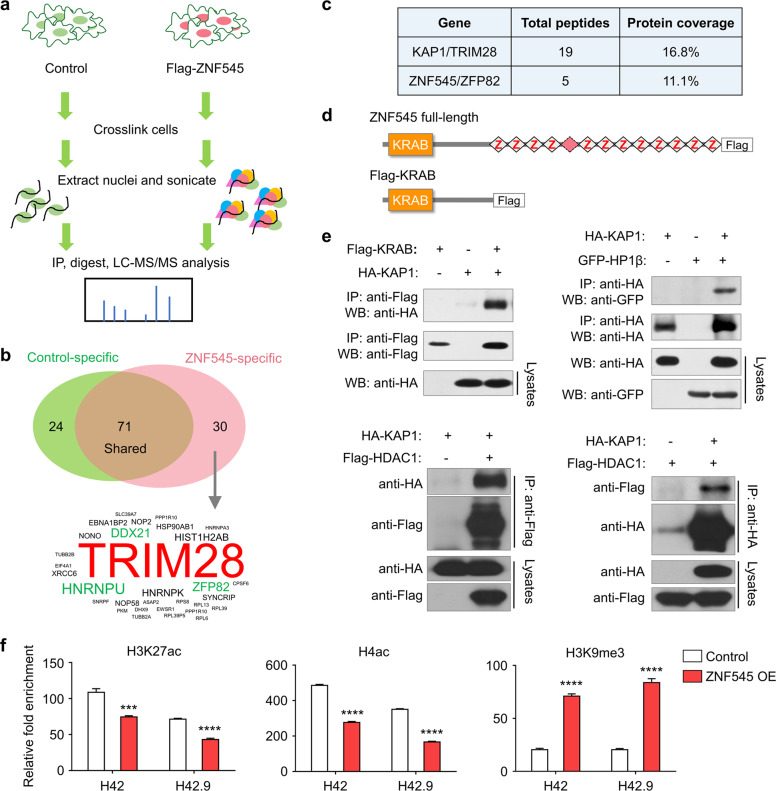
ZNF545 recruits scaffold protein KAP1 to form a transcriptional repressor complex at the rDNA promoter locus. **a** Schematic of the RIME assay. HCT116 cells were transfected with pcDNA3.1 or pcDNA3.1-Flag-ZNF545 and harvested at 24 h post-transfection. ZNF545-containing complex in nuclear extracts was precipitated with anti-Flag antibody followed by LC-MS/MS analysis. **b** Upper: Venn diagram representing the unique and shared identified proteins between RIME experiments for ZNF545-expressing and control group. Based on a threshold of two detected peptides, 30 proteins uniquely identified in ZNF545 RIME were ZNF545-associated proteins while 71 other proteins identified in ZNF545 RIME ware also found in the control group. Lower: Word cloud illustrating ZNF545-associated proteins in HCT116 cells. The word size shows the number of the detected peptides for each ZNF545-associated protein in RIME. **c** The number of detected peptides and protein coverage for KAP1 and ZNF545 in ZNF545 RIME. **d** Schematic representation of Flag-tagged full-length ZNF545 and KRAB domain. **e** Co-IPs showing the physical interactions of KAP1 with the ZNF545 KRAB domain, HP1β, and HDAC1. **f** Relative fold enrichment of histone modification H3K27ac, H4ac, and H3K9me3 at the rDNA promoter determined by ChIP analysis using chromatin prepared from ZNF545-expressing and control HCT116 cells (each group: *n* = 3). ChIP PCR was performed using primer set H42, which amplifies a region ~1 kb upstream of the core promoter region, and primer set H42.9, which amplifies the minimal rDNA promoter region (−57 to +33). All histogram data represent mean ± SD (two-sided Student’s *t* test). ***P < 0.001; ****P < 0.0001.

Next, we sought to identify the potential interaction between ZNF545 and its transcriptional corepressors. We performed rapid immunoprecipitation mass spectrometry of endogenous protein (RIME) profiling assay to probe chromatin-associated protein complex involving ZNF545. Flag-tagged ZNF545 and empty vector overexpressing cells were cross-linked, extracted, and sonicated, followed by anti-Flag pulldown and liquid chromatography with tandem mass spectrometry (LC-MS/MS) analysis (Fig. 4a). LC-MS/MS analysis unraveled 30 unique proteins from the ZNF545-overexpressing group (Fig. 4b; Supplementary Table 2), and KAP1 (also known as TRIM28) was the top enriched target with 19 mapped peptides (coverage: 16.8%) (Fig. 4b,c). KAP1 is a universal corepressor for the KRAB-ZFP family via direct interaction with the KRAB domain of KRAB-ZFPs [5]. Co-immunoprecipitation (co-IP) was next performed to validate the interaction between KAP1 and ZNF545. HA-tagged KAP1 was co-immunoprecipitated with the KRAB domain of ZNF545 by anti-Flag antibody (Fig. 4d, e), inferring that the KRAB domain of ZNF545 was responsible for the recruitment of KAP1. KAP1 serves as a scaffold for corepressors, such as heterochromatin protein 1 (HP1) and histone deacetylases (HDACs) [24, 25]. Co-IP confirmed the strong interactions of KAP1 with HDAC1 and HP1β (Fig. 4e). HDAC1 participate in rDNA silencing through deacetylation of histones, while HP1 is also closely involved in rDNA silencing by binding to repressive histone marker H3K9me3 and leading to heterochromatin formation [2, 26]. Therefore, we examined the effect of ZNF545 on H3K27ac and H4ac, two histone acetylation marks for active gene transcription, as well as repressive H3K9me3 histone mark on the rDNA promoter. Our data indicated that ZNF545 overexpression suppressed H3K27ac and H4ac, while H3K9me3 was induced on the rDNA promoter, as determined by quantitative chromatin immunoprecipitation (ChIP)-PCR (Fig. 4f). Collectively, ZNF545 recruits a repressor complex through KAP1 to suppress rRNA transcription.

### *Znf545* knockout deregulates nucleolus microarchitecture and induces rRNA synthesis

**Fig. 5:**
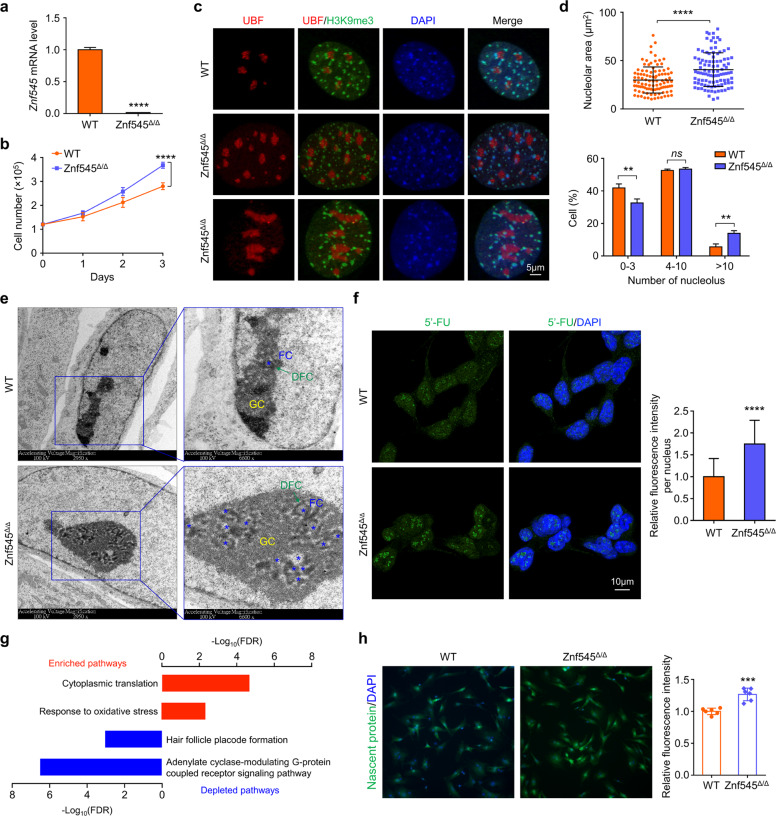
*Znf545* knockout results in the abnormally activated nucleolus, enhanced ribosome biogenesis, and global protein translation. **a** qPCR confirming *Znf545* mRNA expression in *Znf545*^*Δ*/*Δ*^ MEFs (WT: *n* = 5; *Znf545*^*Δ*/*Δ*^: *n* = 4). **b** Cell growth curves of *Znf545*^*Δ*/*Δ*^ and WT MEFs (each time point: *n* = 4; two-way ANOVA). **c** Representative immunofluorescence staining images of the nucleolar marker UBF and heterochromatin marker H3K9me3 in *Znf545*^*Δ*/*Δ*^ and WT MEFs. Patches of heterochromatin are located at the nucleolar periphery. **d** Quantification of the nucleolar area (upper; *n* = 100) and number (lower: WT: *n* = 3; *Znf545*^*Δ*/*Δ*^: *n* = 4) in *Znf545*^*Δ*/*Δ*^ and WT MEFs. **e** Representative electron micrograph of *Znf545*^*Δ*/*Δ*^ and WT MEFs showing nucleolar structure. FC, fibrillar center (blue asterisk); DFC, dense fibrillar components; GC, granular component. **f** Representative immunofluorescence images (left) and quantification (right) of de novo synthesis of pre-rRNA assessed by in situ 5′-FU incorporation into nascent rRNA. 90 nuclei were analyzed in each group. **g** Top Gene Ontology (GO)/biological process (BP) pathways enriched and depleted in *Znf545*^*Δ*/*Δ*^ versus WT MEFs based on SILAC analysis. **h** Representative immunofluorescence images (left) and quantification (right; *n* = 6) of nascent protein synthesis in cultured *Znf545*^*Δ*/*Δ*^ and WT MEFs. Each replicate is the mean fluorescent signal of all the cells in the image. The data (**a**), (**d**), (**f**), (**h**) represent mean ± SD (two-sided Student’s *t* test). Compared with WT MEFs, ns not significant; **P < 0.01; ***P < 0.001; ****P < 0.0001.

The nucleolus is the principal site of ribosome biogenesis, including the synthesis and processing of rRNA. However, CRC cell lines are highly proliferative with deregulated nucleolus [4]. We thus isolated isogenic mouse embryonic fibroblasts (MEFs) from *Znf545*^*Δ/Δ*^ and WT mice to evaluate the effect of ZNF545 loss on the nucleolus (Fig. 5a). Consistent with that in CRC cells, *Znf545*^*Δ/Δ*^ MEFs demonstrated increased proliferation compared to WT MEFs (Fig. 5b). *Znf545*^*Δ*/*Δ*^ MEFs exhibited enlarged nucleolus, with a significant increase in nucleolar area, and increased proportion of cells with multiple nucleolus (number > 10) compared to WT MEFs (Fig. 5c, d), consistent with enhanced nucleolus activity. To gain further insights into microarchitecture changes of nucleolus induced by ZNF545 loss, transmission electron microscopy (TEM) was conducted using thin sections cut through the nucleolus. Three sub-compartments exist within the nucleolus: the fibrillar center (FC) surrounded by dense fibrillar components (DFC), which are embedded in the granular component (GC) [27]. rRNA transcription occurs within FC and pre-rRNA transcripts are processed in DFC, followed by pre-ribosome assembly in GC. As shown in Fig. 5e, WT MEFs have small nucleolus with single FC and few surrounding DFC and GC, indicating a low activity of ribosomal biosynthesis. In contrast, *Znf545*^*Δ*/*Δ*^ MEFs recapitulated the nucleolus phenotype found in fast-growing normal cells and tumor cells with active rRNA synthesis, with DFC forming a large net-like structure intertwined with several to dozens of FCs, which are embedded in a large area of GC (Fig. 5e). To assess nascent rRNA synthesis, the run-on assay was performed by pulsing MEF cells with 5′-fluorouridine (5′-FU), resulting in the incorporation of 5′-FU into nascent pre-rRNA [7, 28]. We observed that *Znf545*^*Δ*/*Δ*^ MEFs exhibited significantly elevated rRNA transcription rate as measured by 5′-FU incorporation levels (Fig. 5f). Enhanced rRNA synthesis was further supported by the increased H3K27ac and H4ac, together with decreased H3K9me3, implying an active chromatin state on the rDNA promoter (Supplementary Fig. S4a).

### *Znf545* knockout promotes protein translation

Ribosome is a ribonucleoprotein complex that functions as the central hub of the translation machinery to translate mRNA into proteins [3]. We, therefore, analyzed the proteomic profiles in *Znf545*^*Δ*/*Δ*^ and WT MEFs using stable isotope labeling by amino acids in cell culture (SILAC) analysis, which relies on the metabolic incorporation of isotopically labeled specific amino acids into cellular proteins [29]. Pathway enrichment analysis of differentially expressed proteins revealed that up-regulated proteins upon knockout of *Znf545* were most significantly enriched in the cytoplasmic translation pathway (Fig. 5g; Supplementary Table 3), which included translation initiation factors (EIF3D and EIF4H), ribosomal proteins from the 40 S/60 S subunits (RPS21, RPS7, RPL22, and RPL10A), and polypeptide chain-releasing factors (GSPT1 and GSPT2), which are involved in translation termination. Western blot and quantitative PCR (qPCR) further confirmed that *Znf545* knockout markedly enhanced the protein expression of EIF3D and RPS21 without obviously affecting their mRNA expression (Supplementary Fig. S4b, c). Hence, *Znf545* knockout coordinately increased rRNA transcription and other essential components of the translational machinery. Consequently, global protein synthesis in *Znf545*^*Δ*/*Δ*^ MEFs was noticeably increased compared to WT MEFs (Fig. 5h). In addition, several key glycolysis-related enzymes were up-regulated in *Znf545*^*Δ*/*Δ*^ MEFs (e.g., PKLR, PYGB, and ALDOC). Indeed, *Znf545*^*Δ*/*Δ*^ MEFs showed increased glycolysis as measured by L-lactate production (Supplementary Fig. S4d). Increased glycolysis would allow increased energy production to support protein translation and cell growth.

### Pol I inhibitor suppressed *Znf545* knockout-induced tumor growth in *Apc*^*Min/+*^ mice

**Fig. 6:**
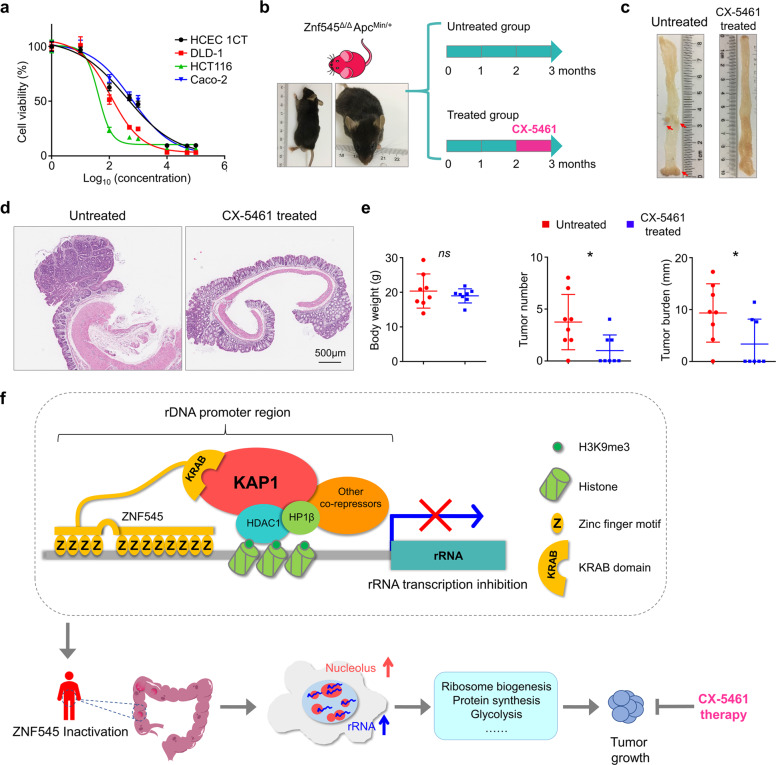
Administration of Pol I inhibitor CX-5461 efficiently suppressed tumor growth in *Znf545*^*Δ/Δ*^*Apc*^*Min/+*^ mice. **a** Drug sensitivity to CX-5461. Cells were treated with CX-5461 for 72 h (0, 10 nM, 100 nM, 500 nM, 1 µM, 10 µM, 50 µM, 100 µM; each concentration: *n* = 5). **b** Scheme for the experimental design of CX-5461 treatment in vivo. Representative photographs (**c**) and hematoxylin and eosin (H&E) staining (**d**) of the intestines from control and CX-5461-treated *Znf545*^*Δ/Δ*^*Apc*^*Min/+*^ mice at 3 months of age. **e** The body weight, colon tumor number, colon tumor burden of control and CX-5461-treated *Znf545*^*Δ/Δ*^*Apc*^*Min/+*^ mice at 3 months of age (each group: *n* = 8). All data represent mean ± SD (two-sided Student’s *t* test). ns, not significant; *P < 0.05. **f** Scheme of the proposed mechanism of malignant transformation by ZNF545 loss.

Our data suggest that CRC cells increased ribosome biogenesis to meet the demand for enhanced protein synthesis and cell proliferation through down-regulating ZNF545. Thus, ZNF545-silenced tumor cells might be more dependent on ribosome biogenesis. In this regard, we treated 3 CRC cells and immortalized normal human colonic epithelial cell line HCEC 1CT with CX-5461, a small molecule inhibitor of Pol I transcription [30–32]. Consistent with our hypothesis, ZNF545-depleted HCT116 and DLD-1 cells were more sensitive to CX-5461 than Caco-2 and HCEC 1CT cells with endogenous ZNF545 expression (Fig. 6a; IC_50_: HCT116: 42.08 nM; DLD-1: 110.5 nM; Caco-2: 586.4 nM; HCEC 1CT: 391.7 nM). We next evaluated whether CX-5461 can be used for the treatment of *Znf545* knockout-induced CRC in mice. CX-5461 was administered orally (50 mg/kg) to *Znf545*^*Δ/Δ*^*Apc*^*Min/+*^ mice every 3 days for 30 days at 2 months old (Fig. 6b). CX-5461 significantly reduced tumor incidence (37.5% (3/8 mice) in the CX-5461 group versus 87.5% (7/8 mice) in the control group; *P* < 0.05), tumor number (*P* < 0.05), and tumor burden (*P* < 0.05) (Fig. 6c–e; Supplementary Table 1), suggesting that CX-5461 inhibited CRC development induced by ZNF545 loss. No significant changes in mice body weight were observed (Fig. 6e), inferring that CX-5461 was well tolerated.

## Discussion

Ribosome biogenesis plays a pivotal role in tumorigenesis by supporting robust protein translation in rapidly proliferating cancer cells. In this study, we identified ZNF545 as a repressor of ribosome biogenesis that suppresses CRC development. Consistent with its functional role as a tumor suppressor, knockout of *Znf545* in mice promotes colorectal tumorigenesis in two CRC models. Mechanistically, we showed that ZNF545 binds to the minimal rDNA promoter and recruits corepressors to mediate repressive histone modifications, leading to suppressed rRNA transcription, ribosome biogenesis and protein translation. Our results suggest that ZNF545 serves as a guardian against dysregulated ribosome activity in colorectal carcinogenesis, and its silencing is pivotal to CRC development.

We investigated the tumor suppressive potential of ZNF545 by first establishing *Znf545* knockout mice. *Znf545* knockout increased the tumor number and tumor load in both genetically- (*Apc*^*Min/+*^) or carcinogen- (AOM/DSS) driven CRC mouse models. Consistent with its tumor suppressive effect in vivo, the ectopic expression of ZNF545 in CRC cell lines suppressed cell growth and induced apoptosis. Collectively, our findings provide evidence that ZNF545 functions as a tumor suppressor in CRC.

rRNA is an essential component of the ribosomes and it is frequently hijacked by cancer cells to boost ribosome biogenesis. Given that ZNF545 functions as a transcription repressor for rDNA, we next elucidated the direct binding of ZNF545 to rDNA and its consequential effect on rRNA-dependent ribosome biogenesis. Several lines of evidence suggest ZNF545 as a critical gatekeeper for preventing excess rRNA transcription: (1) ZNF545 directly binds to rRNA minimal promoter region, the site for Pol I pre-initiation complex formation; [33] (2) either of zinc finger clusters of ZNF545 is targeted to the nucleolus, where they can bind to rDNA to mediate transcriptional repression. Such redundancies represent a “fail-safe” mechanism that ensures the competence of ZNF545; and (3) ZNF545 harbors a KRAB domain, which recruits KAP1, a scaffold for assembling the repressor complex, and thus forming a repressive epigenetic environment on rDNA promoter (Fig. 6f). As a consequence, ectopic ZNF545 expression suppressed rRNA biosynthesis and ribosome biogenesis in CRC cells.

Elevated rRNA transcription following ZNF545 loss has a major effect on the nucleolus, the principal site of ribosome biogenesis. ZN545-null cells demonstrated enlarged nucleolar size, increased nucleolar number and altered nucleolar microarchitecture, reflecting abnormal activation of nucleolus. Disrupted nucleolus homeostasis is increasingly recognized as a hallmark of cancers and a morphological cue for the early diagnosis of precancerous lesions [34]. Apart from supporting elevated cellular turnover in tumors, the nucleolus is functionally important for tumor development, as increased ribosome biogenesis facilitates an altered translation pattern favoring the translation of proto-oncogenes, antiapoptotic factors, and matrix remodeling proteins, thus rendering tumor cells a growth advantage and potential for neoplastic transformation [3, 35]. Mechanistic investigation revealed that boosted ribosome biogenesis caused by ZNF545 loss promoted the expression of essential proteins involved in the translational machinery, coordinately contributing to global protein synthesis. In addition, ZNF545 loss promoted glycolysis by up-regulating rate-limiting enzymes to provide energy for increased protein biosynthesis and cell proliferation. Collectively, our results suggest that ZNF545 loss is a major cause of nucleolar alterations and imbalanced ribosome homeostasis, an indispensable step in tumorigenesis in CRC.

In light of the pro-tumorigenic role of dysregulated ribosome biogenesis, a number of drugs targeting rRNA synthesis have been developed that demonstrated high selectivity for transformed cells [4]. Our data indicated that Pol I inhibitor CX-5461 could selectively kill ZNF545-silenced CRC cells without harming ZNF545 expressing normal colon epithelial cells. In vivo, CX-5461 showed significant efficacy in suppressing the growth of ZNF545-null CRC tumors. CX-5461 is currently undergoing phase I/II clinical trials for advanced hematologic malignancies and triple negative or BRCA-deficient breast cancer and it is safe at doses associated with rapid tumor clearance [32]. Our findings indicate that the targeting of rRNA synthesis might be a feasible strategy for treatment of CRC. In recent years, other anti-tumor mechanisms of CX-5461 independent of rRNA transcription inhibition have been identified, including inducing DNA damage and DNA G-quadruplex formation [36–38]. Further investigation is warranted to assess whether rRNA-independent mechanisms are also involved in the sensitivity of ZNF545-silenced CRC cells towards CX-5461.

In summary, ZNF545 is a tumor suppressor in CRC through transcriptional repression of rRNA transcription. Loss of ZNF545 expression resulted in increased rRNA transcription and ribosome biogenesis, nucleolar abnormality, consequently contributing to colorectal tumorigenesis. Finally, we demonstrate that rRNA biogenesis is a potential vulnerability of ZNF545-null CRC tumors that could be targeted using established rRNA synthesis inhibitors such as CX-5461.

## Materials and methods

### Primary CRC and adjacent normal tissue samples

A total of 143 patients with histologically confirmed CRC who underwent surgery at Prince of Wales Hospital, the Chinese University of Hong Kong, were obtained as described previously [9]. Biopsy samples from primary CRC tumor and adjacent normal were obtained from CRC patients at the time of operation before any therapeutic intervention. All patients provided informed consent for collecting the specimens for study. The study protocols have been approved by the Clinical Research Ethics Committee of Prince of Wales Hospital and the Chinese University of Hong Kong. All patients provided written informed consent for obtaining the study specimens. This study was carried out in accordance with the Declaration of Helsinki of the World Medical Association. In addition, human normal adult colon tissue RNA samples were purchased commercially (Cat. #AM7986, Thermo Fisher Scientific).

### Cell lines

Eight colon cancer cell lines HCT116 (Cat. #CCL-247), HT-29 (Cat. #HTB-38), Caco-2 (Cat. #HTB-37), LoVo (Cat. #CCL-229), SW480 (Cat. #CCL-228), SW620 (Cat. #CCL-227) and LS 180 (Cat. #CL-187) were obtained from the American Type Culture Collection (ATCC). Cell lines were maintained according to protocols from ATCC. The primary immortalized human colonic epithelial cell HCEC 1CT was obtained from Dr. Jerry W. Shay (University of Texas Southwestern Medical Center, USA) and cultured on low oxygen. All cell lines were authenticated by short tandem repeat (STR) every 6 months and tested for mycoplasma contamination using LookOut® Mycoplasma PCR Detection Kit (Cat. # MP0035, MilliporeSigma).

### Generation of constitutive *Znf545*^*Δ/+*^ and *Znf545*^*Δ/Δ*^ mice

*Znf545*-deficient C57BL/6J mice containing a “knockout-first” allele was generated by Beijing Biocytogen Co., Ltd, Beijing, China (Supplementary Fig. S1a) [10]. The “knockout-first” conditional allele was generated by inserting a gene trap cassette through homologous recombination in mouse embryonic stem cells into intron 4 of mouse *Znf545* gene. Floxed conditional *Znf545* allele (*Znf545*^*flox*^) was generated by cross-mating mice harboring “knockout-first” allele with mice expressing Flp (B6.129S4-Gt(ROSA)26Sortm1(FLP1)Dym/RainJ; Beijing Biocytogen Co., Ltd). Constitutive *Znf545* knockout mice (*Znf545*^*Δ/+*^ and *Znf545*^*Δ/Δ*^) were generated by cross-mating *Znf545*^*flox/flox*^ mice with mice expressing the cytomegalovirus (CMV) promoter−Cre (*CMV-Cre*) (B6.C-Tg(CMV-cre)1Cgn/J; Beijing Biocytogen Co. Ltd). Genotyping was performed by PCR of tail-snip DNA using genotyping primers (Supplementary Fig. S1b; Supplementary Table 4). All of the transgenic mice were housed in a pathogen-free barrier environment for the duration of the study at the Department of Laboratory Animal Science at the Army Medical University (Chongqing, China). All procedures were approved by the Animal Ethics Committee of the Army Medical University.

### Generation of colon epithelium-specific *Znf545* knockout mouse

*CDX2-CreER*^*T2*^ transgenic mice was purchased from Jackson Laboratory (Bar Harbor, ME). *Znf545*^*flox/flox*^ mice was bred to *CDX2-CreER*^*T2*^ to generate *Znf545*^*flox/+*^*CDX2-CreER*^*T2*^ and *Znf545*^*flox/flox*^*CDX2-CreER*^*T2*^ mice (colon epithelium-specific *Znf545*^*−/+*^ and *Znf545*^*−/−*^ mice) (Supplementary Fig. S1a, c).

### Generation of *Znf545* deficient MEFs

The isolation of MEFs was performed as described [11]. *Znf545*^*Δ/+*^ and *Znf545*^*Δ/Δ*^ MEFs were isolated from embryonic fetuses after intercross of constitutive *Znf545*^*Δ/+*^ mice. WT MEFs were obtained by cross-mating WT mice. Pregnant mice were sacrificed at 13–14 days post-coitum by cervical dislocation. The uterine horns were dissected out, rinsed and each embryo was separated. The head, tail, all four limbs and red organs were removed. The left tissues were minced, digested with trypsin. After centrifuge, the cell pellet was resuspended in warm DMEM medium supplemented with 10% FBS, 100 µg/ml penicillin, and 50 µg/ml streptomycin sulfate.

### Statistical analysis

Data are presented as mean ± standard deviation (SD). The two-sided Student’s *t* test was used to compare the difference between 2 preselected groups. The chi-square test was used for comparison of incidence. The difference in growth rate between the two groups was determined by repeated measures analysis of variance (ANOVA). Value of *P* < 0.05 was taken as statistical significance.

## Supplementary information

Supplementary Materials and Methods

Supplementary Table 1

Supplementary Table 2

Supplementary Table 3

Supplementary Table 4
