# Supplementary material for: ZNF545 loss promotes ribosome biogenesis and protein translation to initiate colorectal tumorigenesis in mice
Source: Oncogene. 2021 Oct 6;40(48):6590–600. doi: 10.1038/s41388-021-01938-8 (PMC8639438; doi:10.1038/s41388-021-01938-8)
Supplement: Supplementary file 1 — Supplementary Materials and Methods [file 41388_2021_1938_MOESM1_ESM.pdf]

Supplementary Figure Legend

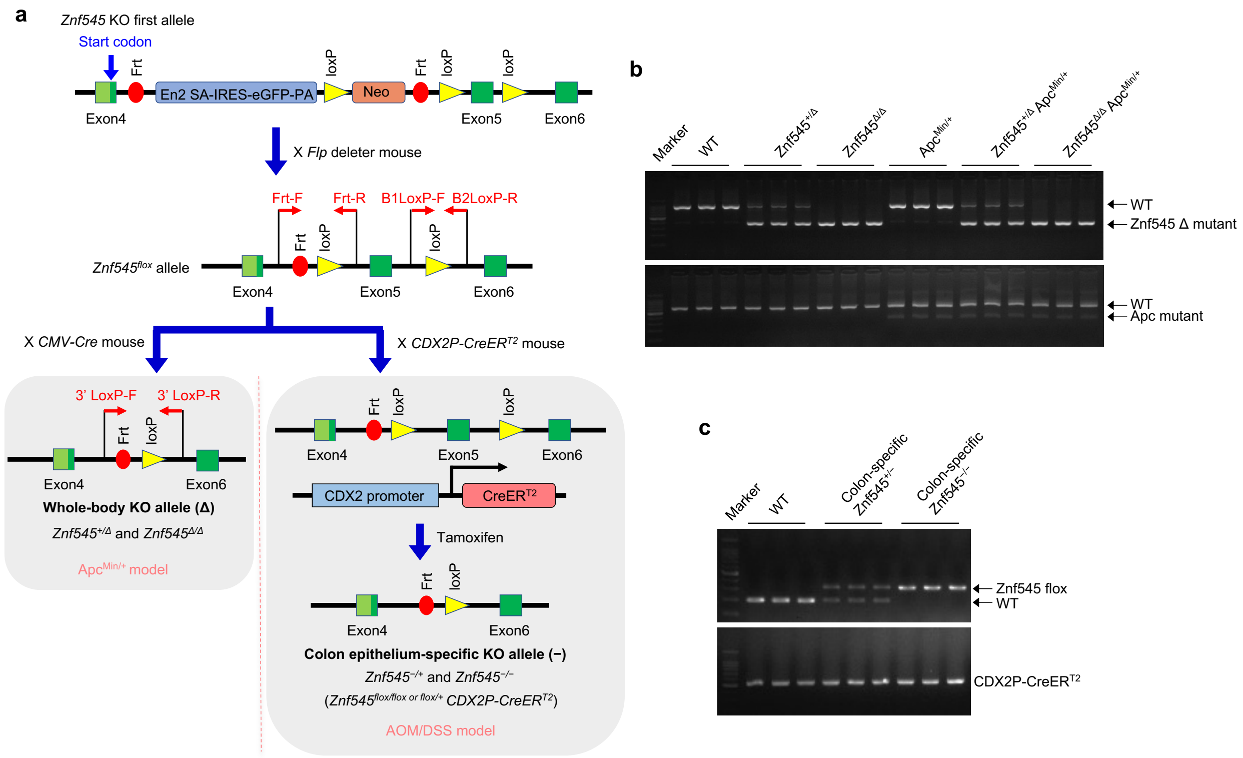

Supplementary Fig. S1: Generation of *Znf545* knockout mice.

a Schematic representation of transgene recombination. The 'knockout-first' conditional allele was generated by inserting a gene trap cassette, consisting of an engrailed 2 splice acceptor (En2 SA)-IRES-eGFP-PA sequence followed by a pGK promoter-neomycin-resistance gene construct, by homologous recombination in mouse embryonic stem cells into intron 4 of mouse *Znf545* gene. Mouse embryonic stem cells derived from C57BL/6N mice containing the correctly targeted *Znf545* locus were injected into blastocysts and transplanted in pseudopregnant mice to generate chimaera mice. Highly chimeric males were mated with C57BL/6N females, and germ line transmission of the targeted allele was confirmed by PCR. The En2 SA-IRES-eGFP-PA and Neo cassettes were removed by flippase (Flp)-mediated excision by crossing with mice ubiquitously expressing Flp, resulting in the pre-conditional floxed *Znf545* allele, in which the critical exon 5 of *Znf545* is flanked by loxP sites. Cre-mediated excision of the floxed exon 5 results in a frameshift and a premature stop codon in exon 6. To generate whole-body *Znf545* knockout mice, germline Cre-mediated excision was performed by cross-mating *Znf545^{flox}* mice with CMV-Cre mice. The inducible conditional knockout of *Znf545* in mouse colon was

generated by breeding *Znf545*^{flox} mice to *CDX2P-CreER*^{T2} mice. The genotype from the mice in the indicated group (**b,c**) were confirmed using RT-PCR of the DNA extracted from the mouse tail. **b** In whole-body *Znf545* knockout (*Znf545*$^{\Delta}$) mice, primers 3' LoxP-F/3' LoxP-R were used to identify deleted exon 5, generating a 367bp PCR product. **c** In colon-specific *Znf545* knockout mice, primers Frt-F/Frt-R were used to identify the floxed exon 5, generating a 320bp PCR product.

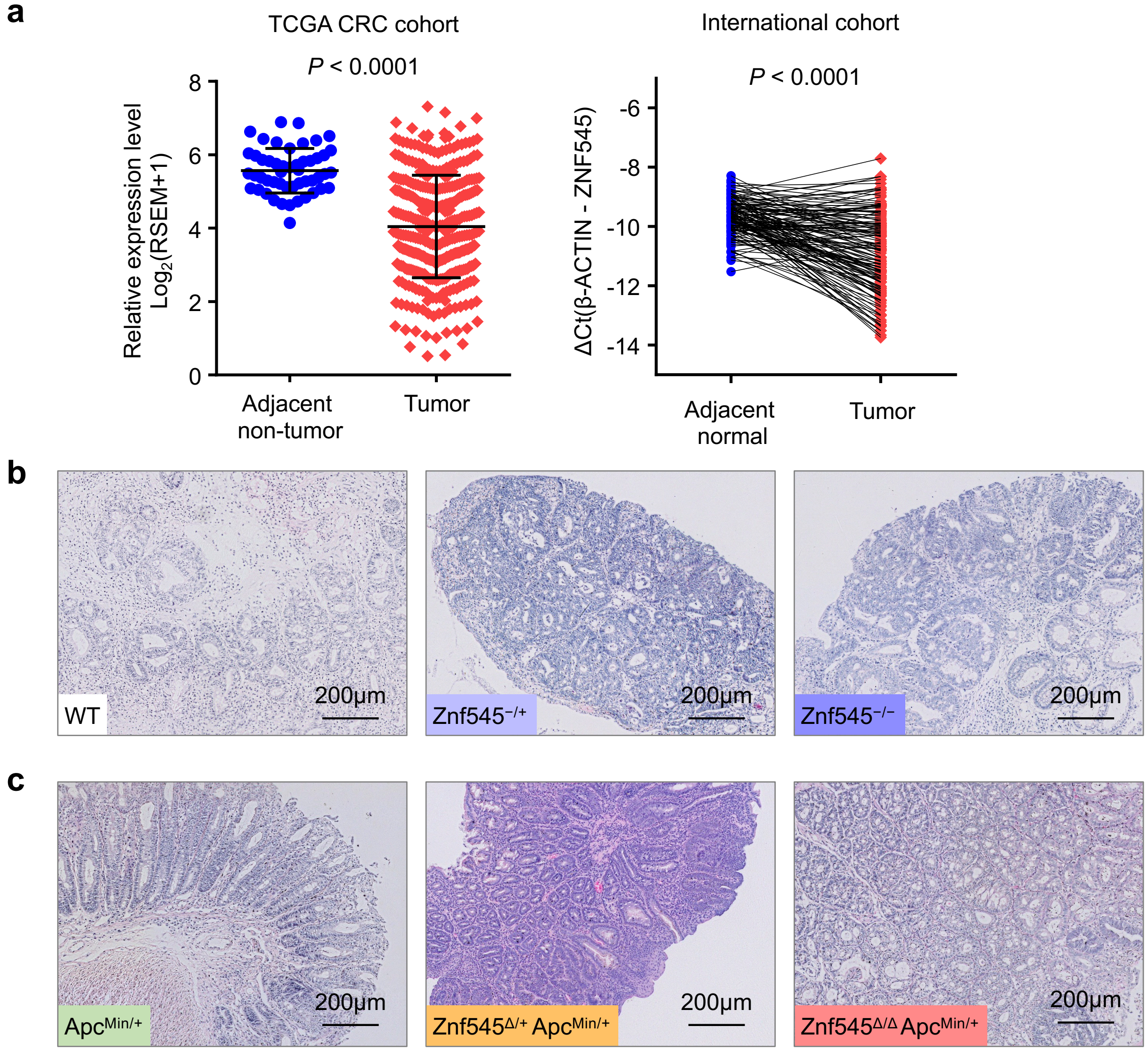

Supplementary Fig. S2: ZNF545 is frequently down-regulated in CRC.

a Expression of *ZNF545* mRNA in the TCGA cohort (non-tumor: n = 50; tumor: n = 380; two-sided unpaired Student's t test) and our cohort (n = 143; two-sided paired Student's t test). **b** Representative H&E staining of colon tumors from WT, colon-specific $Znf545^{-/+}$ and $Znf545^{-/-}$ mice at the experimental endpoint in AOM/DSS model. **c** Representative H&E staining of colon tumors from $Apc^{Min/+}$, $Znf545^{\Delta/+}Apc^{Min/+}$, $Znf545^{\Delta/\Delta}Apc^{Min/+}$ mice at 3 months of age in $Apc^{Min/+}$ mice model.

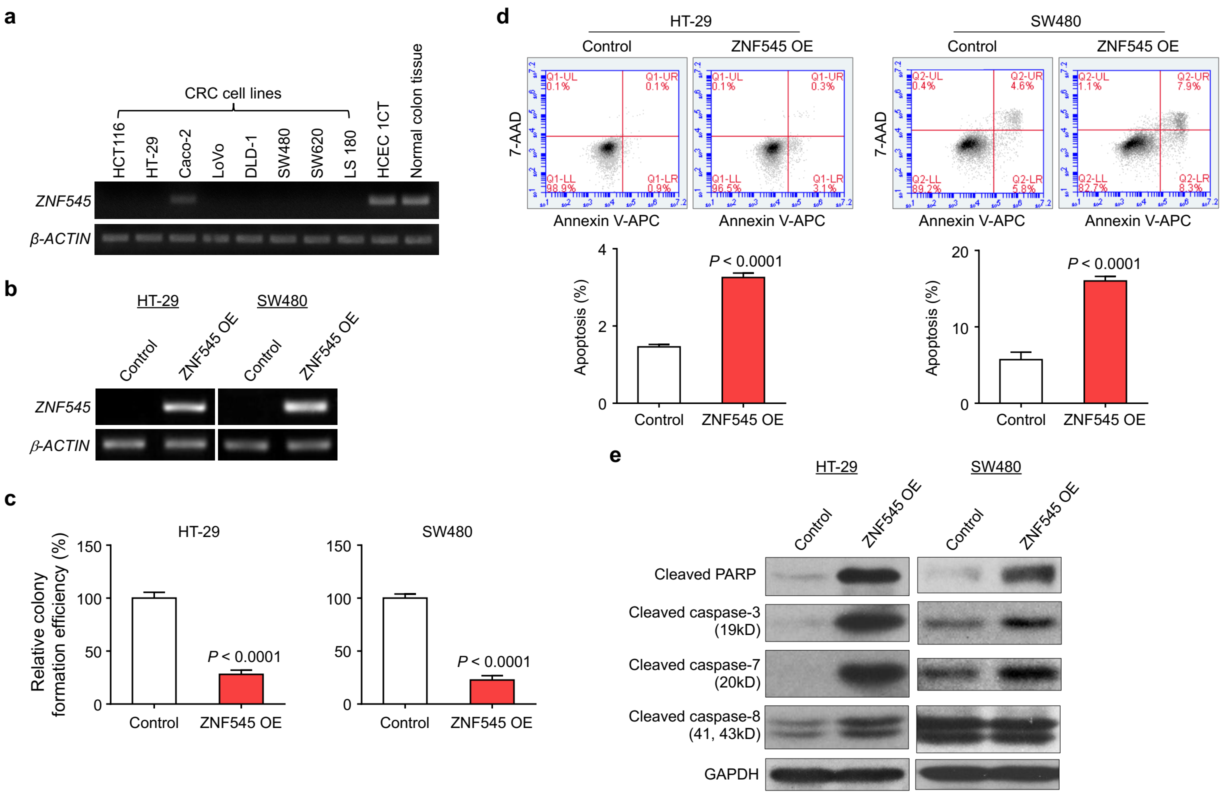

Supplementary Fig. S3: ZNF545 inhibits CRC cell growth and induces cell apoptosis.

a Expression of endogenous *ZNF545* mRNA in CRC cell lines, immortalized normal human colonic epithelial cell line HCEC 1CT and normal human colon tissue. **b** *ZNF545* mRNA expression upon ectopic expression of ZNF545. **c** Effect of ZNF545 overexpression on colony formation (each group: n = 3). **d** Cell apoptosis assessed by flow cytometry analysis of Annexin V-APC and 7-AAD double-staining (each group: n = 3). **e** Western blot of cleaved caspase-3, -7, -8 and PARP. All histogram data represent mean ± SD (two-sided Student's t test).

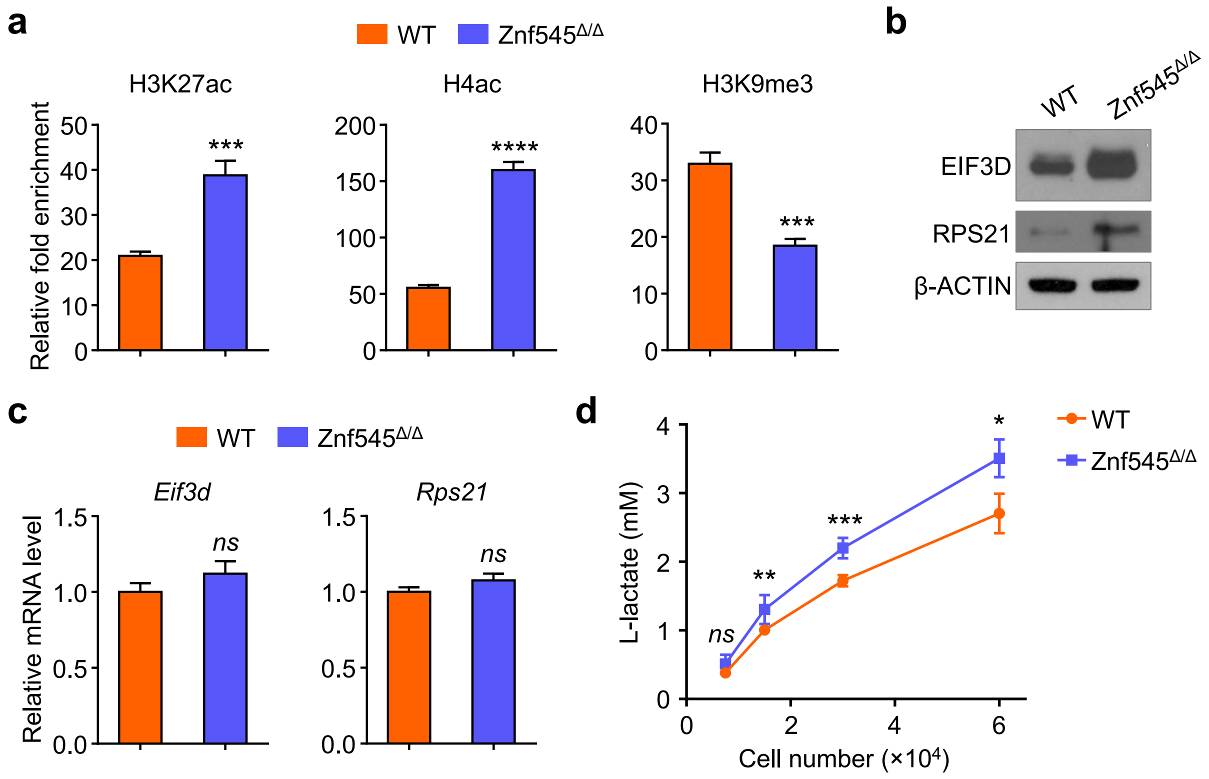

Supplementary Fig. S4: Phenotypic and functional comparison of *Znf545*$^{Δ/Δ}$ and WT MEFs.

a Relative fold enrichment of histone modification H3K27ac, H4ac and H3K9me3 at the mouse core rDNA promoter region determined by ChIP analysis using chromatin prepared from *Znf545*$^{Δ/Δ}$ and WT MEFs (each group: n = 3). Western blot (**b**) and qPCR (**c**; each group: n = 3) analysis of EIF3D and RPS21 expression in *Znf545*$^{Δ/Δ}$ and WT MEFs. **d** Enzymatic quantification of L-lactate accumulation in the supernatant of cultured *Znf545*$^{Δ/Δ}$ and WT MEFs 24 hours after seeding. Glycolysis levels were quantified over a range of cell densities (each condition: n = 8). All data represent mean ± SD (two-sided Student's t test). Compared with WT MEFs, ns, not significant; *$P < 0.05$; **$P < 0.01$; ***$P < 0.001$; ****$P < 0.0001$.

Supplementary Tables

Supplementary Table 1. Summary of tumor number, tumor burden and body weight in mice models.

Supplementary Table 2. Summary of RIME analysis.

Supplementary Table 3. Summary of SILAC analysis.

Supplementary Table 4. Summary of primer sequences and antibodies.

Supplementary Materials and methods

Plasmids and drugs

The full-length ZNF545 cDNA was amplified and cloned into the pCDNA3.1+ expression vector (Cat. #V79020, Thermo Fisher Scientific) and lentiviral vector pLVX-Puro (Takara Bio USA).[1] The rDNA promoter serial deletion constructs were generated by PCR amplification using pHrD-IRES-Luc as the template with the reverse primer, 5'-GGTACCTATCGATAGAGAAATGTTCTGGC-3' and the following forward primers: 5'-TGACCAGAGGGCCCCG-3' (pHrD-IRES-Luc-deletion-1); 5'-TCCCGCTCTGGAGACACG-3' (pHrD-IRES-Luc-deletion-2). Flag-tagged KRAB-Hand 1, KRAB-Hand 2, KRAB and KRAB-deletion were constructed by fusion PCR amplification using pcDNA3.1-Flag-ZNF545 as the template as indicated in the figure. GFP-Hand 1 and GFP-Hand 2 were constructed by fusion PCR amplification using pcDNA3.1-Flag-ZNF545 and pEGFP-C1 (Takara Bio USA) as the templates as indicated in figure. GFP-tagged HP1β (Cat. #17651) and Flag-tagged HDAC1 (Cat. # #13820) were purchased from Addgene. HA-tagged KAP1 was cloned into the pCDNA3.1+ expression vector using plasmid template (Flag-TRIM28, Cat. #124960) from Addgene. CX-5461 (Cat. #S2684) was purchased from Selleck Chemicals.

RNA extraction and qPCR

Total RNA was extracted using Trizol reagent (Cat. # 15596018, Thermo Fisher Scientific). cDNA was synthesized from total RNA using High-Capacity cDNA Reverse Transcription Kit (Cat. # 4368814, Thermo Fisher Scientific). Primer sequences are listed in **Supplementary Table 4**.

Immunofluorescence staining

Cells were seeded on coverslips in a 6-well plate. Cells were fixed with methanol or 4% paraformaldehyde and permeabilized. Nonspecific binding sites were blocked with 1% BSA/PBS. Primary antibodies were applied at room temperature for 2 h. After PBS wash, secondary antibodies with desired fluorescence probes were applied. Then cells were washed and mounted with ProLong™ Gold Antifade Mountant with DAPI (Cat. #P36962, Thermo Fisher Scientific). The information of antibodies was listed in **Supplementary**

Table 4.

Dual-luciferase reporter assay

HCT116 cells were plated in 24-well and co-transfected with 200 ng pHrD-IRES-Luc or deletion variants, 5 ng *Renilla* luciferase vector pRL-CMV, 800 ng pcDNA3.1 or pcDNA3.1-Flag-ZNF545 or ZNF545 variants. Cells were harvested 24 hours post-transfection and luciferase activities were analyzed by the dual-luciferase reporter assay system (Promega). Reporter activity was normalized to the control Renilla. Experiments were repeated in triplicate.

Apoptosis assay

Apoptosis was determined by dual staining with APC Annexin V (Cat. #550474, BD Biosciences) and 7-amino-actinomycin (7-AAD) (Cat. #559925, BD Biosciences). Cells were harvested, stained and analyzed by flow cytometry according to the manufacturer's instructions (BD Biosciences). Sample fluorescence of 10,000 cells was analyzed using FACSCalibur System (BD Biosciences). The relative proportion of Annexin V-positive cells was counted as apoptotic cells.

EMSA

Nuclear proteins were extracted from HCT116 cells using NE-PER™ Nuclear and Cytoplasmic Extraction Reagents (Cat. #78833, Thermo Fisher Scientific) according to the manufacturer's instructions. The 5'-biotinylated oligo probes were obtained from Integrated DNA Technologies and annealed (**Supplementary Table 4**). EMSAs were performed using LightShift™ Chemiluminescent EMSA Kit (Cat. # 20148, Thermo Fisher Scientific). 300 fmol of each biotin-labeled probe were incubated with 20 μg nuclear proteins in EMSA binding buffer for 20 min at room temperature. Competition reactions were performed by adding 100-fold unlabeled probes. For achievement of a supershift, 10 μg anti-Flag antibody was added.

Co-IP assay

Co-IP analyses were carried out as previously described.[1] Briefly, total protein was

extracted from HCT116 cells (~5 × 10^6/reaction) in Radio Immuno Precipitation Assay buffer (RIPA; 50 mM Tris-HCl at pH 8.0, 150 mM NaCl, 1.0% NP-40, 0.5% sodium deoxycholate, 0.1% SDS, 1×protease inhibitor mixture). Lysates were pre-cleared and immunoprecipitation was performed using anti-Flag M2 antibody (Cat. #F1804, MilliporeSigma), anti-HA antibody (Cat. #sc-805, Santa Cruz Biotechnology) and Protein G-agarose beads overnight at 4°C. The supernatant was removed and the protein G beads-protein complex were washed with RIPA buffer. Finally, the precipitated proteins were denatured and evaluated by Western blot.

ChIP assay

ChIP experiments were performed as previously described.[2] Briefly, cells were crosslinked with formaldehyde. The crosslinking reaction was stopped by adding glycine and collected for nuclei protein extraction. Chromatins from extracted crosslinked nuclei were sheared by sonication and precipitated with antibodies to acetyl-Histone H4 (H4ac) (Cat. #06-866, MilliporeSigma; Cat. # 39925, Active Motif), Histone H3 (acetyl K27) (H3K27ac) (ab4729, Abcam) and Histone H3 (trimethyl K9) (H3K9me3) (ab8898, Abcam) followed by capturing immunoprecipitated proteins/DNA complex with protein-G-magnetic beads. Equal numbers of cells (1 ~ 2 × 10^7) were used for each immunoprecipitation. After reversal of the cross-link and digestion of proteins with proteinase K, the immuoprecipitated DNA was isolated by phenol/chloroform/isoamyl alcohol method. The fold enrichment was calculated relative to the background detected with non-specific IgG for rDNA promoter primer as indicated previously (**Supplementary Table 4**).[3]

***Apc$^{Min/+}$* mouse model**

Znf545$^{Δ/+}$ mice and *Apc$^{Min/+}$* mice (strain: T001457; Nanjing Biomedical Research Institute of Nanjing University) were used to generate *Znf545$^{Δ/+}$Apc$^{Min/+}$ and Znf545$^{Δ/Δ}$Apc$^{Min/+}$* mice. For CX-5461 treatment, *Znf545$^{Δ/Δ}$Apc$^{Min/+}$* mice were randomly divided into two groups before drug treatment. At 3 months, mice were sacrificed and screened for intestinal tract tumors. The investigators were not blinded concerning group allocation.

AOM/DSS mouse model

7-week old colon-specific *Znf545*$^{-/+}$, colon-specific *Znf545*$^{-/-}$ and WT (*CDX2P-CreER*^{T2}) mice were intraperitoneally (i.p.) injected with tamoxifen dissolved in corn oil (100 mg/kg) for four consecutive days. Two weeks after first tamoxifen dose, mice were injected i.p. with 10 mg/kg AOM (MilliporeSigma) and further given three cycles of 2% DSS for 7 days in weeks 3, 6 and 9, and killed in week 14 (4 weeks after the end of the third DSS cycle).[4] Intestine examination, tumor measurement and histology inspection were performed. The investigators were not blinded concerning group allocation.

Mouse intestinal tumor analysis

Mice were sacrificed by CO_2, and their intestines were removed and flushed with PBS. Intestines were opened longitudinally and analysed for number, location, and size of tumors. Tumor size was calculated by the mean of its length, width, and height. Tumor burden was the sum of all the tumour size per mouse.[4] Fresh tissues were fixed in 4% formalin, embedded in paraffin, followed by H&E staining for histology examination. The pathology characteristics of tumors were analyzed with help from certified pathologists.

RIME assay

RIME was performed following a protocol described previously.[5] Briefly, HCT116 cells were transfected with pcDNA3.1 or pcDNA3.1-Flag-ZNF545 and crosslinked with formaldehyde at 24 h post-transfection. After sonication, nuclear extracts were incubated with Flag antibody (Cat. #F1804, MilliporeSigma)-bound beads (Cat. #10003D, Thermo Fisher Scientific). After extensive washing step, the beads-bound proteins were digested using trypsin-containing buffer overnight followed by LC-MS/MS analysis.

Protein synthesis assay

Protein synthesis assay was conducted using Click-iT™ Plus OPP Alexa Fluor™ 488 Protein Synthesis Assay Kit (Cat. #C10456, Thermo Fisher Scientific) following the manufacturer’s protocol. Mouse cells were cultured on pre-coated glass cover slides. Briefly, cells were treated with 20 μM Click-iT® OPP working solution for 30-min incubation under culturing conditions. The medium was then removed, and the cells were

fixed and permeabilized with 3.7% formaldehyde and 0.5% Triton X-100 (in PBS), respectively. After washing, cells were incubated with a freshly prepared Click-iT® Plus OPP reaction cocktail for 30 min and washed with Click-iT® Reaction Rinse Buffer. Finally, cells were counter stained with HCS NuclearMask™ Blue Stain working solution and sent for imaging and analysis. All the pictures were acquired using identical settings and analyzed using ImageJ. Each replicate is the mean fluorescent signal of all the cells in the picture. For comparison, fluorescence intensities from experimental groups were normalized to that from control.

Glycolysis assay

Glycolysis was assessed using Glycolysis Cell-Based Assay Kit (Cat. #600450, Cayman Chemical) following the manufacturer's protocol. Cells were seeded in DMEM containing 10% FBS in a 96-well plate. Next day medium was replaced with 100 μl of DMEM containing 1% FBS. Cells were grown for 18 h before L-lactate detection. The standard was prepared by serial dilution. 10 μl of the standards and supernatant from each well of the cultured cell plate were used for analysis.

Run on assay

Pulses of 5′-FU (Cat. #F5130, MilliporeSigma) was administered to a final concentration of 2 mM as described previously.[6] After 10 min of incubation, cells were fixed with 3.7% paraformaldehyde in PBS. The incorporation of 5′-FU into nascent RNA was detected with anti-BrdU antibody (Cat. #B8434, MilliporeSigma).

SILAC analysis

Isotopically labeled lysine ($^{13}C_6$ $^{15}N_2$ L-Lysine-2HCl (heavy)), isotopically labeled arginine ($^{13}C_6$ $^{15}N_4$ L-Arginine-HCl (heavy)), unlabeled L-Lysine-2HCl (light), L-Arginine-HCl (light), Dialyzed FBS and DMEM medium were purchased from N-Cell Technology. Supplemented media "Light" and "Heavy" were freshly prepared according to the manufacturer's protocol. *Znf545*$^{\Delta/\Delta}$ and WT MEF cells were split using one containing heavy and one containing light SILAC medium, respectively. After cells underwent at least five cell doublings, labeled cells were extracted with NitroExtraTM (Cat. #PEX-001-

250ML, N-Cell Technology). Protein were precipitated with 1:3 cold acetone and re-suspended in 8M urea. Proteins are reduced, alkylated and diluted to 2M urea followed by trypsin digestion. Digestion was performed in 100mM triethylammonium bicarbonate (pH 8, Cat. #T7408, Sigma) and 37°C for 18 h. Digested proteins were desalted by C18 column.

Fractionation of peptides was conducted by strong cation exchange (SCX) liquid chromatography. Samples were re-suspended in SCX Buffer A (10mM KH_2PO_3, 20% ACN, pH 2.7). SCX is performed with PolySULFOETHYL A™ (200x4.6-mm, 200A) column using step gradient (0-10 mins: 0%B, 29 mins: 15%B, 44 mins: 45%B, 46-53 min: 100%B) of Buffer A and Buffer B (10mM KH_2PO_3, 20% ACN, 0.6M KCl, pH 2.7) and a flow rate of 1min/ml. In total 53 x 1ml fractions were collected and then combined into 10 fractions. Each fraction was desalted with ZipTip (Cat. #ZTC18S960, MilliporeSigma) and dried in spin vacuum for LC-MS/MS analysis.

The sample was analyzed by nanoLC-MS/MS using an Eksigent ekspert™ nanoLC 425 system coupled to AB Sciex TripleTOF® 6600 System. Peptide was trapped (ChromXP nanoLC Trap column 350 µm id x 0.5 mm, ChromXP C18 3 µm 120Å) and eluted at a flow rate of 300nL/min into a reverse phase C18 column (ChromXP nanoLC column 75 µm id x 15 cm, ChromXP C18 3um 120Å) using a linear gradient of acetonitrile (3-36%) in 0.1% formic acid with a total runtime of 120 mins including mobile phase equilibration. Mass spectra and tandem mass spectra were recorded in positive-ion and “high-sensitivity” mode with a resolution of ~35,000 full-width half-maximum. The nanospray needle voltage was typically 2,300 V in HPLC-MS mode. For collision induced dissociation tandem mass spectrometry (CID-MS/MS), the mass window for precursor ion selection of the quadrupole mass analyzer was set to ± 2 m/z. The precursor ions were fragmented in a collision cell using nitrogen as the collision gas. Advanced information dependent acquisition (IDA) was used for MS/MS collection on the TripleTOF 6600 to obtain MS/MS spectra for the 20 most abundant and multiple charged (z = 2, 3 or 4) following each survey MS1 scan allowing typically for 250 msec acquisition time per each MS/MS. Dynamic exclusion was set for 30 secs after 2 repetitive occurrence.

Raw data files were converted to Mascot Generic Format (MGF) and mzXML format using OpenMS. The MGF files were searched against the SwissProt and common MS contaminant database using Mascot (Matrix Science) Software. The tolerance for MS1 and MS2 error was 50 ppm and 50 mmu respectively. Caramidomethylation (+57 Da) is added as fixed modification while Label:13C(6) 15N(2)(K), Label: 13C(6) 15N(4) (R), and Oxidation (M) was added as variable modifications. A maximum of 2 trypsin miss cleavages were allowed. Peptides were assumed to have a charge of 2+, 3+ or 4+. The instrument type was chosen as ESI-QTOF. The mass input was assumed to be monoisotopic mass. Decoy database was used for the estimation of false discovery rate (FDR). Ratio of each protein was given by geometric mean of the protein ratios measured from all replicates. Two-tailed student's t-test was performed to test the significance of protein ratios and Benjamini-Hochberg multiple hypothesis test correction is employed to correct the P-values. The protein ratios of $P < 0.05$ with 5% FDR correction were filtered as differential proteins for following analyses.

TEM

The cell suspension was fixed in equal volume of 2.5% glutaraldehyde in cacodylate buffer (0.1 M sodium cacodylate-HCl buffer pH 7.4). After centrifuge, cells were washed with cacodylate buffer with 0.1 M sucrose once and with cacodylate buffer once. Cells were fixed in 1% osmium tetroxide (OsO_4) in cacodylate buffer and washed with cacodylate buffer. After centrifuge, cell pellet was resuspended in equal volume of pre-warmed 2% agar solution for gel block. Ultra-thin sections of 100 nm thickness (gold colour) with glass/diamond knife and ultramicrotome were cutted. Sections were mounted on the 150 mesh hexagonal copper grids and stained with 2% aqueous uranyl acetate and Reynold's lead citrate. The Philips CM100 TEM in the University of Hong Kong was used for digital imaging.

References

1 Wang S, Cheng Y, Du W, Lu L, Zhou L, Wang H *et al.* Zinc-finger protein 545 is a novel tumour suppressor that acts by inhibiting ribosomal RNA transcription in gastric

cancer. *Gut* 2013; **62**: 833–841.

2 Zhang Y, Wang S, Kang W, Liu C, Dong Y, Ren F *et al.* CREPT facilitates colorectal cancer growth through inducing Wnt/β-catenin pathway by enhancing p300-mediated β-catenin acetylation. *Oncogene* 2018; **37**: 3485–3500.

3 Grandori C, Gomez-Roman N, Felton-Edkins ZA, Ngouenet C, Galloway DA, Eisenman RN *et al.* c-Myc binds to human ribosomal DNA and stimulates transcription of rRNA genes by RNA polymerase I. *Nat Cell Biol* 2005; **7**: 311–318.

4 Neufert C, Becker C, Neurath MF. An inducible mouse model of colon carcinogenesis for the analysis of sporadic and inflammation-driven tumor progression. *Nat Protoc* 2007; **2**: 1998–2004.

5 Mohammed H, Taylor C, Brown GD, Papachristou EK, Carroll JS, D'Santos CS. Rapid immunoprecipitation mass spectrometry of endogenous proteins (RIME) for analysis of chromatin complexes. *Nat Protoc* 2016; **11**: 316–326.

6 Torrano V, Navascués J, Docquier F, Zhang R, Burke LJ, Chernukhin I *et al.* Targeting of CTCF to the nucleolus inhibits nucleolar transcription through a poly(ADP-ribosyl)ation-dependent mechanism. *J Cell Sci* 2006; **119**: 1746–1759.
